# Perceived barriers to effective use of telehealth in managing the care of patients with cardiovascular diseases: a qualitative study exploring healthcare professionals’ views in Jordan

**DOI:** 10.1186/s12913-023-09368-w

**Published:** 2023-05-08

**Authors:** Ibtisam A. Alarabyat, Nezam Al-Nsair, Intima Alrimawi, Nabeel Al-Yateem, Raed Mohammad Shudifat, Ahmad Rajeh Saifan

**Affiliations:** 1 Nursing College, Applied Science Private University, Amman, Jordan; 2 Nursing College, The University of Jordan, Amman, Jordan; 3 College of Nursing, Xavier University, Cincinnati, OH 45207 USA; 4 School of Nursing, Georgetown University, Washington DC, USA; 5 Department of Nursing, College of Health Sciences, University of Sharjah, Sharjah, United Arab Emirates; 6 Charles Sturt University, Bathurst, Australia; 7 Adult Health Nursing Department, Faculty of Nursing, Mu’tah University, Mu’tah, Jordan; 8 Nursing College, Applied Science Private University, P.O. Box: 541350, Amman, 11937 Jordan

**Keywords:** Telehealth, Cardiovascular disorders, Remote care, Jordan, Qualitative research

## Abstract

**Background:**

The use of telehealth in the management of care and care delivery has been increasing significantly during the COVID-19 pandemic. Telehealth is an emerging technology used to manage care for patients with cardiovascular diseases (CVDs) in Jordan. However, implementing this approach in Jordan faces many challenges that need to be explored to identify practical solutions.

**Purpose:**

To explore the perceived challenges and barriers to using telehealth in managing acute and chronic CVDs among healthcare professionals.

**Methods:**

A qualitative, exploratory study was conducted by interviewing 24 health professionals at two hospitals in different clinical areas in Jordan.

**Results:**

Several barriers were reported by participants that affected the utilization of telehealth services. The barriers were categorized into the following four themes: Drawbacks related to patients, Health providers’ concerns, Procedural faults, and telehealth To complement the service only.

**Conclusions:**

The study suggests that telehealth can be instrumental in supporting care management for patients with CVD. It means that understanding the advantages and barriers to implementing telehealth by the healthcare providers in Jordan can improve many aspects of the healthcare services for patients with CVD within the healthcare settings in Jordan.

## Background

Since the COVID-19 pandemic in 2019, telehealth has grown more crucial for managing healthcare, including cardiovascular care, and has taken the lead and been at the core of healthcare delivery [1]. In 2020, most healthcare systems had to change how they provide care in response to pandemics [2, 3].

Around the world, including in countries like Jordan and Turkey in the Middle East, the number of CVD diagnoses is steadily increasing [4]. By utilising telehealth treatments, early detection and reducing CVD risk factors can be achieved [5–7]. Additionally, telehealth offers the possibility for remote monitoring and self-management of numerous CVD risk factors, making it an efficient and promising tool for containing various aspects of CVDs [8]. Linked technologies have the potential to dramatically change the healthcare sector by reducing the death rate among patients with CVDs and raising the standard of care given to patients and their relatives [9].

However, several obstacles to telehealth’s successful implementation concluded that additional data and evidence are required regarding telehealth benefits [8, 10]. For instance, Mitchell-Gillespie, Hashim [8] concluded that further evidence is needed to support the benefits of telehealth. Furthermore, because many of them are mainly unregulated and not supported by evidence, there are worries about their usage in healthcare delivery. Moreover, there are problems with the application and validity of the findings and limited sample sizes in the existing telehealth research.

In the middle eastern region, the progress and adoption of telemedicine were evaluated, and the associated barriers were discussed in a recent systematic review by Al-Samarraie, Ghazal [11]. Within this systematic review, around 43 peer-reviewed articles from 2010 to 2020 were reviewed for this research. The findings showed that the progress in the implementation of telemedicine is relatively slow and varied in many countries of the region. Cultural, financial, organizational, individual, technological, legal, and regulatory factors have significantly contributed to the slow adoption of telemedicine in the medical field. However, previous reviews didn’t consider the COVID-19 period when Jordan increasingly adopted telehealth. The review found resistance among healthcare professionals and patients, along with poor infrastructure, inadequate funding, inadequate computer systems, and lack of information technology training, are among the factors hindering implementation.

In Jordan, there is an apparent shortage of expertise and information on telehealth applications for patients with CVDs [12, 13]. More aspects of the adoption and utilization of mobile health in the context of refugees in Jordan were studied [12, 13]. The evidence from these studies found some challenges, including the absence of internet connectivity and the required technical know-how to utilize the application. Similar findings were highlighted by Mitchell-Gillespie and his colleagues, who explained that the short-term success in telehealth adoption was observable [8]. However, several obstacles were found, including poor infrastructure, a lack of necessary skills, and cultural beliefs concerning care delivery care. Based on the effectiveness of several telehealth programs in Jordan, investigators claimed that a comprehensive national telehealth project was required, particularly in light of the COVID-19 pandemic [5, 14]. The current study adds to another study that investigated the experiences of healthcare professionals and patients with cardiovascular diseases (CVD) regarding telehealth utilization [6]. This qualitative study aimed to explore the perceived challenges and barriers to using telehealth in managing acute and chronic CVDs among healthcare professionals.

## Aim of the study

The study aims to explore the views of healthcare professionals regarding the barriers to telehealth interventions in Jordan, specifically in cardiovascular disease management.

## Methodology

### Design

The design and methods of the current study are similar to a previous study aimed at exploring the perspectives of CVD patients and health professionals on the importance of telehealth in managing CVD health problems [6]. A qualitative research approach was judged appropriate for this study because it provides an in-depth understanding of research phenomena. Using this methodology, it is possible to examine opinions and experiences and gather rich evidence from those who have experienced the implementation of telehealth in their practice [15, 16].

### Participant selection and recruitment

A purposive sampling technique was used to recruit a total of twenty-four participants. The sample consisted of twenty-four healthcare professionals with experience helping patients with CVDs and willing to participate in individual virtual or face-to-face interviews. The interviews were continued until reaching the point of data saturation. This point is reached when data obtained from the research process does not generate new themes or when the participants in the research do not contribute new information [17].

### Settings

The settings for this study were two major hospitals located in Amman, the capital of Jordan. These institutions provide the services of a telephone conversation or videoconference to facilitate access for patients who have difficulty getting to the sites due to distance or the cost of travel. For the previous considerations, these two hospitals were purposively chosen in the current study.

### Ethical considerations

Institutional Review Board (IRB) approval was obtained from the Research and Ethical Committee at the Applied Science Private University (2020-2021-2-12), Abdali Research and Ethics Committee (2,021,700,001) and Prince Hamza Hospital Ethics Committee (5729). The participants provided informed consent and were assured of their right to refuse to participate or withdraw from the study. Each participant completed a signed consent form; more information was provided by giving them an information sheet. Additionally, anonymity and secrecy were upheld by using Alphanumeric codes to secure the identity of research participants and storing the data in password-protected folders. All the procedures in this study that involved human participants were carried out following the ethical standards of the institutional and national research committee and with the 1964 Helsinki declaration and its later amendments or comparable ethical standards. All methods were carried out under relevant guidelines and regulations.

### Data collection

Semi-structured interviews were used to collect data from participants in the study settings. A 60-minute interview was conducted with each participant. An interview schedule was used to facilitate the interview process and provide consistency in generating data from the respondents (See Table 1 for the interview study guide questions). These questions were generated from the comprehensive literature review. With the participants’ consent, interviews were recorded using a smartphone app, and the transcriptions were verbatim. Additionally, using interview probes allowed the researcher to be flexible and explore unanticipated topics while still adhering to the original interview questions.

**Table 1:** Interview schedule

**The interview questions**: • What do you think is the appropriate means of enhancing the management of cardiovascular diseases in your population? • What are your experiences concerning the reliance on telehealth in a medical context? • What are your concerns regarding the reliance on telehealth to manage cardiovascular diseases? • What do you consider the disadvantages of relying on telehealth in managing cardiovascular diseases? • Would you participate again in these telehealth visits? For what reasons? • Is there anything else you would like to share with me today about your experience with telehealth?	

### Data analysis

The interviews were fully transcribed verbatim. Participants’ demographic information, characteristics, transcriptions, and field notes were uploaded into NVivo 12 PLUS software for analysis. The thematic analysis method developed by [18] was used to analyze the data. Each line of the transcripts was read over several times, and any first thoughts or intriguing details of the material were documented. Key characteristics of the data were given codes, or “nodes”, in NVivo. To analyze the original codes and group them under more salient themes, the study team convened multiple times and worked collaboratively.

### Trustworthiness of the study

The investigators followed a method used by Lincoln & Guba [19] to enhance the trustworthiness of the data during the data collection and analysis process. The technique suggested four criteria to assure trustworthiness: transferability, credibility, confirmability, and dependability.

Credibility was assured by collecting field notes, reflective journals, and verbatim transcripts; Transferability was improved and maintained by the in-depth nature of the interviews and rich and comprehensive data descriptions. Seven participants reviewed their transcripts and made any required adjustments to ensure confirmability. Additionally, until everyone in the study team was satisfied with the results, each theme was discussed several times. Finally, dependability was guaranteed using qualitative data analysis software (NVivo) to evaluate the data and maintain a systematic audit trail. In addition, the triangulation of methods by combining several perspectives of health providers, bracketing techniques, participant validation, and the study protocol helped improve the research validity and reliability.

## Results

### Participants characteristics

A total of 24 health professionals were interviewed. The sample included seven cardiologists, three internists, and fourteen nurses. Most of these professionals had experiences in outpatient clinics and medical and surgical departments. The health professionals were selected because they have experience in telehealth, including communication with patients, coordinating their appointments, explaining some instructions etc. The sample included healthcare professionals who have experience in helping patients with CVDs. Including healthcare professionals with different specialities in this study helped obtain multiple views of the research topic.

A sample of healthcare providers with a minimum of 11 years of experience participated in the study. Most of these professionals were between 34 and 58 years, and just a small percentage of respondents worked for the government (see Table 2).

**Table 2:** Healthcare provider participant characteristics

Participant	Age	Gender	Current workplace	Primary medical specialty	Period of practicing (years)
1.	58	Male	Private	Cardiologist	> 20
2.	35	Male	Public	Nursing	11–15
3.	37	Male	Public	Nursing	11–15
4.	44	Male	Public	Nursing	> 20
5.	57	Male	Public	Internal medicine	> 20
6.	35	Female	Private	Nursing	11–15
7.	35	Female	Public	Nursing	11–15
8.	57	Male	Private	Cardiologist	> 20
9.	37	Male	Private	Cardiologist	11–15
10.	35	Male	Private	Cardiologist	11–15
11.	34	Female	Public	Nursing	11–15
12.	42	Male	Private	Nursing-Quality	11–15
13.	56	Male	Private	Cardiologist	> 20
14.	44	Male	Private	Cardiologist	16–20
15.	55	Male	Private	Cardiologist	> 20
16.	45	Female	Private	Internal medicine	16–20
17.	55	Male	Public	Nursing	> 20
18.	36	Male	Public	Nursing	11–15
19.	34	Male	Public	Nursing	11–15
20.	39	Male	Public	Nursing	11–15
21.	38	Male	Private	Nursing	11–15
22.	36	Female	Private	Nursing	11–15
23.	38	Female	Public	Nursing	11–15
24.	43	Male	Public	Internal medicine	16–20

### Perceived barriers to telehealth

The results showed several drawbacks that were perceived as barriers to the utilization of telehealth in Jordan. These barriers were categorized into the following four main themes (see Table 3):

**Table 3:** The main derived themes and subthemes

Themes	Sub-themes
Perceived barriers to telehealth	Drawbacks related to patients
	Drawbacks related to health providers
	Procedural drawbacks
	Treatment process drawbacks

#### Drawbacks related to patients

There were some drawbacks to using telehealth related to patients. The first is improper use by patients and low awareness of appropriate use. Some patients use this service to exploit the remote checkup to pretend they are sick or send fake tests and checkups to get excuses for their workplaces. Regarding this, one of the health professionals (1) stated:

*“I believe the patient is lying or telling me: ‘I am tired. Would you mind telling me I am ill so I will not go to work?’.” [P1]*

Low awareness is also prevalent among patients about how to use the program. This is related to the nature of the program, which has a lot of medical information that patients may misunderstand and cause a risk to their lives. One of the providers (6) said:

*“The patient may misunderstand what the provider says when we give advice, and the patient does other things due to a misunderstanding.” [P6]*

The findings showed that some patients might be over-reliant on the program and believe there is no need to see the physician in person, which is unavoidable in the case of CVDs. This was found in one of the providers (5) who declared that:

*“Literally 90% of people don’t care about any need in their presence, a waste of time and responsibility. A patient may come to me after two years and tell me he has spoken to me earlier.” [P5]*

The findings uncovered that some of the patients have some fear of using technology, especially for serious diseases. As a result of introducing a new way of doing things, many patients resisted adopting the new program. In this vein, one of the health professionals (3) remarked:

*“Many patients still have some fear of using new technology. Most patients come to the clinic to see the physician in person and do not accept the change. Many patients have to sit with them for a full session to convince them of the idea of ​​telehealth.” [P10]*

The above concerns seem important when implementing telehealth in patient care management. It may be advisable to consider a hybrid system in the initial stages of deployment to familiarize service providers and users with telehealth systems and what to expect from such care delivery.

#### Health providers concerns

Like patients, service providers may also misuse the telehealth system, such as ignoring reading notes on the system and unnecessary referral to consultants based on a preliminary remote diagnosis. For example, one of the providers (5) said:

*“The only problem I have with young physicians is that they do not read notes… patients’ physicians. As a medical consultant, I have to write notes. Due to this, we have some problems, including the patient’s return to the physician for failing to read the notes.” [P5]*

Some health professionals have issues with being able to be contacted by patients on a 24-hour basis, which causes an invasion of their privacy. One of the service providers (7) remarked that:

*“The disadvantage for physicians is the lack of privacy since patients can talk to them anytime. Physicians must respect their time since patients can invade our private lives anytime.” [P7]*

Lastly, the findings revealed that the culture of Jordanian people, especially in rural areas, prefers those female patients to be treated by female physicians and nurses, which poses the challenge of the lack of female healthcare professionals able to provide telehealth services. One of the providers (4) mentioned:

*“It is difficult because only professional men work in the telehealth system, but it would be better if it is a female for a female like me.” [P4]*

#### Procedural faults

The telehealth clinics in remote areas lacked some services related to CVDs and, at the same time, lacked some essential devices. For example, regarding the lack of some services, one of the providers (5) commented:

*“Yes, for example, catheters are not available to them. Whether a heart patient needs catheterization or not. However, suppose patients have a problem with the electrical heart, whether it is acceleration or slowing down of the heartbeat. In that case, An ECG is done for them, and we give them an appointment at the Electrocardiogram Clinic at Prince Hamzah Hospital.” [P5]*

It was explained that telehealth services are limited to a few clinics and hospitals in Jordan. Some participants mentioned that they are facing problems with having longer wait time for appointments, and the need to book advance appointments have been noted by one of the providers (4) as follows:

*“The patient must show up, follow up, or schedule an appointment. I told you we worked in telehealth clinics two days a week. Due to the pressure on Prince Hamza and the corona crisis, it is now one day. They reduced the number of patients and prolonged the booking time.” [P4]*

Due to the long wait time, the analysis documented that some nepotism (the local system of informal professional relationships and activities known as “*wasta”*) was used to speed up some patients’ appointments. One of the providers (4) stated that explicitly alluded to this:

*“Some of our people have flaws in their morals and behavior. There is a kind of wasta [nepotism] in treating patients, but I do not follow this.” [P4]*

Finally, most participants pointed out that the system they are using faces many failures that hinder workflow and cause patient delays. For instance, one of the providers (5) remarked that:

*“Technological things often disrupt our work, such as the Internet and devices breaking down.” [P5]*

#### A complimentary service only

Most participants agreed that telehealth could be effective only for patient follow-up and monitoring their status and medical outcomes. It could not be a complete substitution for a face-to-face meeting. Regarding this, one of the providers (24) said:

*“We cannot say that this is better than this. However, this service must be activated for those patients who need to be tracked down and seen by a physician without going to the hospital.” [P24]*

Interestingly, two health professionals explained that it is challenging to depend on other health professionals to perform diagnostic procedures and physical examinations. They had fears regarding the responsibilities of diagnosing critical diseases and life-threatening conditions based on judgements from other colleagues. These professionals showed that telehealth is not suitable for all diseases. In this line, one of the providers (22) said:

*“How I can depend on other colleagues to diagnose a patient with chest pain or having shortness of breath. This is a life-threatening condition, and I might be accountable if I misdiagnose the patient.”. [P22]*

## Discussion

The findings revealed that telehealth programs in Jordan are facing several challenges, which may primarily be related to the immature implementation on the ground and the intrinsic inability of telehealth solutions to treat all relevant CVDs or stages of therapy. Telehealth faced strong resistance from some participants due to their latent inertia and familiarity with traditional modes of treatment, which they perceive to be more thorough and safer or convenient for them. Such resistance may be related to the fear of adopting telehealth as new technology. This is not surprising, as most literature regarding the adoption of new technologies reveals a significant reluctance among users to undertake and facilitate change and fear among a smaller but substantial proportion. Researchers in these fields have emphasized the importance of understanding human behaviors related to technology to achieve successful technology adoption [10, 20].

However, the findings also showed a growing acceptance rate among patients experiencing telehealth solutions for the first time and a high willingness to recommend them to others. These results are consistent with previous findings. Telehealth is more likely to be accepted by patients if they have used it or learned about it before being referred Woo and Dowding [21], [22]. Patients with no experience with telehealth might become less resistant to telehealth use if they are more aware of its benefits, such as time and cost savings [23]. In Jordan, the high cost incurred by patients is due to treatment expenses, follow-up visits, and associated expenses and efforts to travel from rural and faraway areas to specialized clinics. These findings echo several studies on many illnesses. Clearly, cost and effort are the primary disadvantages of traditional treatment.

Telehealth also has disadvantages, such as improper use and low awareness of proper usage by patients. The health professionals explained that some patients use remote diagnosis to pretend to be sick and get a report not to go to work. Patients are also not aware of how to use the program. This is because the program contains much medical information that patients may misunderstand and put their lives at risk. Other studies reported similar behavioural responses during the pandemic in patients utilizing remote diagnosis [24, 25]. For instance, Balkhi, Nasir [24] found that those unaware of the illness were more likely to pretend to be ill and avoid attending work or school.

Healthcare providers are confronting their challenges with telehealth deployment [26]. For example, some physicians ignore notes on the system and refer patients to consultants based on a preliminary remote diagnosis [27]. In addition, telehealth caused a problem for physicians due to constant access by patients and intrusion into physicians’ private lives. This finding reverberated what other researchers found, as they argued that there is a problem of overreach and burnout when access to healthcare personnel is granted [28–30].

An additional perceived barrier is related to the conservative culture of Jordanians, whereby most female patients prefer to be treated by female healthcare professionals. This is not feasible in many contexts, as most consultants and specialists in heart disease are male. Literature related to Arab and Muslim societies has thoroughly examined these religious and cultural factors to determine the gender preference for receiving treatment from the same gender [31, 32].

Moreover, the findings uncovered that the telehealth centres specialized in cardiovascular interventions lack some services and devices. The deployment of this service in Jordan is minimal due to the government’s financial limitations and the consideration of telehealth as a less-priority, premature, or even experimental service among decision-makers and other stakeholders, including healthcare professionals and patients. According to Muflih, Al-Azzam [33], many community pharmacists are still unprepared to embrace new technologies such as telehealth. Patients still struggle with long-time appointment booking, even when using telehealth. Likewise, nepotism was noted to result in favoritism in the services patients received. Many studies have pointed out the role of this cultural issue in Jordanian society. Al-Twal and Aladwan [33] asserted that *wasta* (nepotism) is widely used in Jordan, and favoritism has been socially sanctioned as an acceptable practice. The findings of this study are consistent with the results of a study conducted by Bieber and Weiner [34]. They found that patients and providers may be frustrated and encounter lower-quality outcomes when telehealth systems are poorly designed or have delayed connections [10].

The findings showed that telehealth in CVD patients is a complicated intervention process that carries risks and has limited scope for follow-up. The increased complexity of CVD might make it slightly more difficult for telemedicine to effectively use in primary care for CVD patients than other service users Harky, Adan [35]. This complexity stems from telehealth interventions in CVD patients entailing particular risks, especially when the system is left to individual judgments.

Additionally, policymakers and healthcare providers need to collaborate more closely and find more effective ways of working together in order to make the policies more efficient. Establishing a dedicated department or committee to regulate telehealth programs is necessary. This issue aligns with what Shachar, Engel [36] advocated by creating a third regulatory path to improve telehealth implementation.

## Strengths and Limitations

The main strength of the study is that the study explored the perspectives of both the healthcare provider and the patient as users toward the use of telehealth for the management of CVDs to provide a broad understanding of telehealth. The healthcare providers had a wide variety of experiences with the health services in Jordan. However, the study could have included other healthcare providers involved in telehealth, such as technicians and pharmacists. It is of note that the sample size of this study was small, and it was conducted in two hospitals in one city in Jordan, which limits the ability to generalize the finding of this study. However, this study revealed in-depth information about the topic of interest that might be transferable to other similar contexts.

## Implications in practice

The study’s findings suggest a great potential to improve the effectiveness of healthcare management using telehealth for patients with CVDs in Jordan. It is recommended that more planning and work is needed in Jordan at the government policy level for successful implementation and adoption. Telehealth provides a flexible and affordable option for patients and healthcare providers; however, more education and training are needed for the end-users to be used at the total capacity. This study can be used in teaching and training as a practical tool for practitioners, policymakers, and students interested in telehealth [22].

The study’s findings contribute to the existing body of literature by adding new perceptions to previous studies and can add to the body of knowledge about telehealth in Jordan and globally to help establish a model of care that can improve the delivery of care in Jordan and the surrounding region. Furthermore, the results can be used for future studies to build on the body of knowledge in this field of research. Additional studies on telehealth that focus on practice policies, adaptation to new technologies, and social influence are recommended to promote adoption and awareness among Jordanian citizens.

## Data Availability

The data is available in the form of audiotapes and transcripts. All data generated or analysed during this study are included in this article. All the data are saved in a secured computer. The datasets used and/or analysed during the current study are available from the corresponding author upon reasonable request.
